# Association Between Birth Timing and Early Clinical Outcomes in Very Preterm Infants: A Retrospective Cohort Study

**DOI:** 10.3390/jcm15218282

**Published:** 2026-10-24

**Authors:** Muhammet Asena, Duygu Tunçel, Leyla Şero, Nilüfer Okur

**Affiliations:** 1 Department of Pediatrics, Istanbul Medipol University, Istanbul 34214, Turkey; 2 Clinics of Neonatology, Department of Pediatrics, Gazi Yasargil Training and Research Hospital, Diyarbakır 21090, Turkey; duygu.tuncel1@saglik.gov.tr (D.T.);; 3 Department of Pediatrics, Gazi Yasargil Training and Research Hospital, Diyarbakır 21090, Turkey; leyla.sero@saglik.gov.tr

**Keywords:** preterm infants, birth timing, off-hours birth, neonatal mortality, intraventricular hemorrhage, neonatal intensive care unit, prematurity outcomes

## Abstract

**Background:** The effect of birth timing on outcomes in very preterm infants remains controversial. This study evaluated the association between birth timing and early morbidity and mortality in infants born before 32 weeks of gestation. **Methods:** In this retrospective single-center cohort study, inborn infants born at <32 weeks’ gestation between January 2021 and May 2023 were categorized as in-hours (weekdays 08:01–16:00) or out-of-hours (weekdays 16:01–08:00, weekends, official holidays). The primary outcome was a composite of early mortality (≤72 h) or severe intraventricular hemorrhage (grades III–IV). Multivariable logistic regression adjusted for baseline confounders (Model 1); delivery-room resuscitation, a potential mediator, was added in Model 2. **Results:** Of 363 infants, 160 (44.1%) were born in-hours and 203 (55.9%) out-of-hours. The composite outcome occurred in 19.7% versus 12.5% (*p* = 0.091; Model 1 aOR 1.46, 95% CI 0.77–2.77). Overall mortality (32.5% vs. 21.3%, *p* = 0.023), early mortality (8.4% vs. 2.5%, *p* = 0.022), delivery-room resuscitation (56.7% vs. 36.9%, *p* < 0.001), and respiratory distress syndrome (69.0% vs. 55.6%, *p* = 0.012) were higher out-of-hours. Out-of-hours birth was associated with higher mortality in Model 1 (aOR 1.88, 95% CI 1.11–3.20), but the estimate was attenuated and imprecise in Model 2 (aOR 1.58, 95% CI 0.91–2.75). **Conclusions:** Out-of-hours birth was not significantly associated with the composite primary outcome but was associated with higher crude and baseline-adjusted mortality. Because delivery urgency, illness severity, and staffing at delivery were incompletely measured, residual confounding cannot be excluded.

## 1. Introduction

Preterm birth, defined as birth occurring before 37 weeks of gestation, remains one of the leading causes of neonatal morbidity and mortality worldwide [1]. The health problems faced by preterm infants are serious and often lead to complications such as respiratory distress syndrome (RDS), intraventricular hemorrhage (IVH), and long-term neurodevelopmental disorders [2]. To improve outcomes, several strategies have been implemented, including optimization of antenatal care, use of antenatal corticosteroids, advances in neonatal intensive care, and supportive interventions aimed at both short-term stabilization and long-term neurodevelopmental outcomes [3,4].

The timing of birth has been proposed as a potential determinant of maternal and neonatal outcomes [5]. Several studies have investigated whether births occurring during nighttime hours, weekends, or off-duty periods are associated with worse clinical outcomes, a phenomenon often referred to as the “off-hours” or “weekend effect” [6,7]. Potential explanations include variations in staffing levels, provider experience, workload, and availability of specialized interventions during different shifts [8]. However, the impact of birth timing remains controversial, as some studies have demonstrated increased mortality and morbidity during off-hours, whereas others have reported no significant differences when standardized care protocols and adequate staffing are maintained.

Interventions performed in the first minutes of life, particularly in infants born at less than 32 weeks of gestation—one of the most vulnerable populations—can substantially affect short- and long-term outcomes. Some studies suggest that births at night or during off-peak hours may be associated with an increased risk of adverse outcomes, including severe IVH, bronchopulmonary dysplasia (BPD), and retinopathy of prematurity, indicating that staffing per patient during off-hours may be important [6,7,9]. In one retrospective study, night-shift births were significantly associated with a higher risk of low Apgar scores, early neonatal death, and fresh stillbirths compared with the morning shift [6]. In a large multicenter cohort of more than 47,000 very low birth weight infants, birth during off-peak hours, particularly overnight, was independently associated with an increased risk of severe IVH and adverse composite outcomes, even after adjustment for perinatal and hospital-level factors [9].

Most evidence regarding the “off-hours effect” comes from high-income countries and large neonatal networks where the practice of having neonatologists on-site around the clock is common [7,9]. Data from tertiary care centers in middle-income countries remain limited; in these settings, while pediatricians and residents are physically present, neonatologists typically provide consultation via telephone during nights and weekends [6,10]. In such environments, various mechanisms may plausibly contribute to the off-hours effect: the experience of the team receiving the infant in the delivery room (initial stabilization minutes may be managed by less experienced staff); obstetric staffing and decision-making processes that can influence the timing and course of emergency deliveries; and workload (situations where fewer staff members are responsible for a larger number of patients and simultaneous admissions) [11]. Therefore, determining whether these structural differences lead to poorer clinical outcomes for extremely preterm infants is a significant question for centers with similar staffing models.

In this study, we aimed to investigate the relationship between the timing of birth and clinical outcomes in preterm infants born before 32 weeks of gestation at our institution—a regional tertiary care center in a middle-income country where neonatologists provide on-site care during regular working hours but offer telephone consultation outside these hours. We hypothesized that off-hours births would be associated with a higher risk of the primary composite endpoint, which included early mortality (within the first 72 h) or severe intraventricular hemorrhage (IVH); overall mortality and major neonatal morbidities were considered as pre-specified secondary endpoints.

## 2. Materials and Methods

### 2.1. Study Design and Setting

This retrospective single-center cohort study was conducted at the tertiary-level Neonatal Intensive Care Unit (NICU) of Gazi Yaşargil Training and Research Hospital, Diyarbakır, Turkey. The study included preterm infants born before 32 completed weeks of gestation between January 2021 and May 2023.

In the NICU, neonatal care is provided by neonatology specialists on weekdays and non-official holidays between 08:00 and 16:00. Outside regular working hours, a pediatric specialist and a pediatric resident are on duty; neonatology specialists provide consultation by phone and attend the hospital when required. Every weekend and on official holidays, neonatology specialists attend the unit to conduct ward rounds for all patients and plan treatment and follow-up together with the on-call team.

According to local NICU guidelines, one nurse was assigned to every three to four patients. This nurse-to-patient ratio remained unchanged during and outside working hours; however, outside regular hours there were no dedicated nurses responsible for supply management, supervision, catheter care, or postoperative care.

Two main groups were established. Group 1 (in-hours) consisted of infants born during regular working hours (08:01–16:00 on weekdays), when the full multidisciplinary team was present. Group 2 (out-of-hours) included infants born between 16:01 and 08:00 on weekdays and those born on weekends and official holidays. Births occurring exactly at 08:00 were assigned to the out-of-hours group and those at 16:00 to the in-hours group; these boundary cases were individually reviewed.

The study was performed in line with the Declaration of Helsinki. The protocol was approved by the Clinical Ethics Committee of Diyarbakır Gazi Yaşargil Training and Research Hospital (date: 2 December 2026; approval number: 62). Given the retrospective design, the requirement for individual informed consent was waived by the ethics committee.

### 2.2. Study Population

All infants admitted to our Neonatal Intensive Care Unit (NICU) during the study period were evaluated for eligibility. The inclusion criteria were as follows: gestational age of less than 32 weeks; delivery at Gazi Yaşargil Training and Research Hospital; and admission to the NICU following birth. Infants who died in the delivery room prior to NICU admission were excluded from the study.

**Figure 1:**
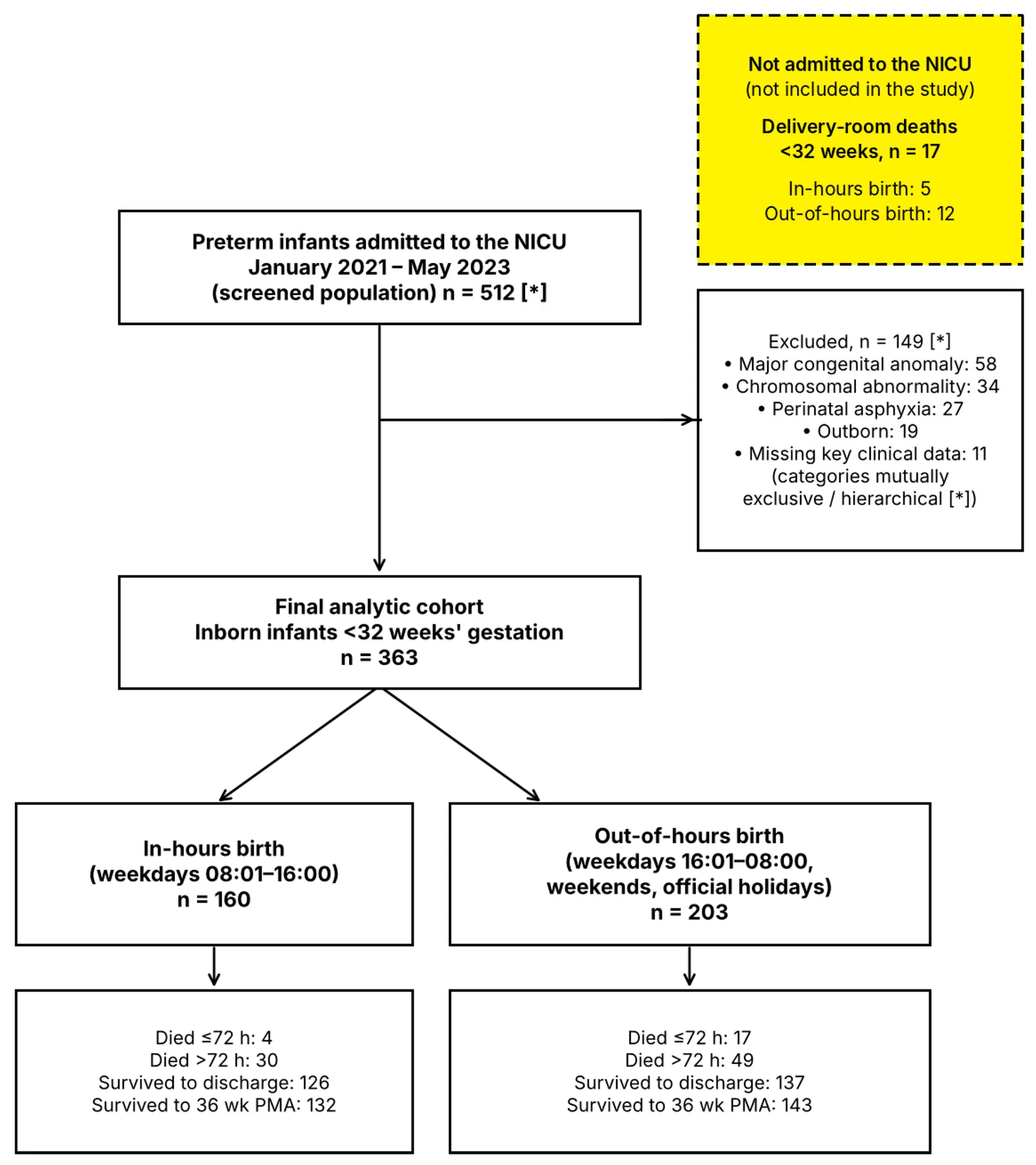
Flow diagram of study participants. NICU, neonatal intensive care unit; PMA, postmenstrual age. * excluding patients with incomplete data who have been transferred to another center.

The exclusion criteria were as follows: major congenital anomalies (structural anomalies independently determine neonatal prognosis and represent a distinct clinical population); chromosomal anomalies; perinatal asphyxia; out-of-hospital birth (due to potential variations associated with transport during the initial stabilization phase); and missing essential clinical data. Perinatal asphyxia was determined based on discharge diagnoses recorded by the attending neonatologist and was operationally defined as the presence of clinical signs of encephalopathy and/or multi-organ dysfunction in conjunction with an umbilical cord or first-hour arterial pH < 7.00 and/or a base deficit ≥12 mmol/L. Since complications during childbirth may be associated with both the timing of delivery and mortality, the exclusion of infants with perinatal asphyxia could have introduced selection bias. Specifically, if asphyxia occurs more frequently in births taking place outside of regular working hours, the exclusion of these cases may have led to an underestimation of the association between the timing of delivery and adverse outcomes. As clinical data for the excluded infants were not collected, it was not possible to conduct a sensitivity analysis that included these cases; this is acknowledged as a limitation. Infants meeting more than one exclusion criterion were counted once under the first applicable criterion in the hierarchy in question (Figure 1).

### 2.3. Definition of Exposure: Birth Timing

In-hours birth was defined as weekdays between 08:01 and 16:00, and out-of-hours birth as weekdays between 16:01 and 08:00, weekends, and public holidays. This classification reflects institutional staffing patterns. Births at the exact boundary times (08:00 and 16:00) were individually reviewed and classified as described above.

### 2.4. Data Collection

Maternal and neonatal data were obtained from the electronic medical records of Gazi Yaşargil Training and Research Hospital. Maternal variables included maternal age, gravidity, mode of delivery (cesarean section [C/S] or normal spontaneous vaginal delivery), gestational diabetes, chorioamnionitis, preeclampsia, early membrane rupture, preterm premature rupture of membranes (PPROM), and antenatal steroid exposure. Additional obstetric variables obtained from the records included multiple pregnancy, placental abruption, and placenta previa. Information regarding planned versus emergency delivery, spontaneous versus medically indicated preterm birth, specific indications for delivery, and acute fetal distress (e.g., abnormal fetal heart rate monitoring) could not be analyzed because it was not consistently recorded in the electronic records. Antenatal steroid exposure was defined as a documented complete course. Binary clinical variables were coded as “present” only when documented in the medical records; cases without such documentation were considered “absent.”

Neonatal variables included birth weight (g), gestational age (weeks), sex, and 5 min Apgar score. The 1 min Apgar score was not included as a primary variable because it primarily reflects the need for immediate resuscitation and is considered less predictive of long-term outcome than the 5 min Apgar score, a validated predictor of neonatal morbidity and mortality in preterm infants [12]. The need for delivery-room resuscitation (intubation, cardiopulmonary resuscitation [CPR], and epinephrine administration) was recorded separately. Umbilical cord venous blood gas values (pH and base deficit) were collected.

### 2.5. Outcomes

The single primary outcome was a composite result comprising early mortality (death within the first 72 h after birth) or severe intraventricular hemorrhage (IVH; grades III–IV) diagnosed at any time during the neonatal intensive care unit (NICU) stay. This composite outcome was selected because both events occur during the same critical early postnatal period, share common pathophysiological mechanisms (including hemodynamic instability and impaired cerebral autoregulation), and have been used as a composite endpoint in previous studies evaluating birth timing and staffing in extremely preterm infants [9,13]. Overall mortality was defined as death due to any cause prior to discharge from the NICU. Prespecified secondary outcomes were: overall in-hospital mortality, early mortality, severe IVH, RDS, air leak syndrome, hemodynamically significant patent ductus arteriosus (hsPDA), necrotizing enterocolitis (NEC; modified Bell stage ≥II), moderate-to-severe BPD, periventricular leukomalacia (PVL), feeding outcomes, duration of invasive mechanical ventilation (IMV), and length of hospital stay. IVH of any grade, pulmonary hemorrhage, surfactant administration, inotrope use during the first week, early- and late-onset sepsis, fungal sepsis, and retinopathy of prematurity (ROP) were analyzed as exploratory outcomes. Retinopathy of prematurity screening, diagnosis, follow-up, and treatment were performed in accordance with the 2021 Turkish Neonatal Society and Turkish Ophthalmological Association ROP Guidelines. Infants born at <34 weeks of gestation or with a birth weight ≤1700 g, as well as clinically high-risk preterm infants, were eligible for screening. Retinal examinations were performed by an ophthalmologist experienced in ROP screening using binocular indirect ophthalmoscopy. Initial examinations were scheduled at 30–31 weeks of postmenstrual age for infants born before 27 weeks of gestation and at four weeks of postnatal age for those born at ≥27 weeks. Subsequent examinations were scheduled according to retinal findings and disease severity, generally at intervals of 1–3 weeks, with more frequent assessments when indicated. Outcomes assessable only in surviving infants were analyzed in the relevant surviving infants: moderate-to-severe BPD in infants alive at 36 weeks of postmenstrual age (PMA) (the composite outcome of death or moderate-to-severe BPD was reported for the entire cohort); ROP in infants surviving beyond 28 days; PVL in infants surviving beyond 72 h; time to achieve full enteral and full oral feeding and duration of hospital stay in infants discharged alive; and duration of invasive mechanical ventilation (IMV) in infants receiving IMV (both in the overall group and among survivors).

### 2.6. Definitions

IVH was graded according to the Papile classification [14]. Cranial ultrasonography was performed by a radiologist according to the unit protocol: on days 1, 3, 7, 14 and 30 in infants born before 28 weeks of gestation, and on days 3, 7, 14 and 30 in those born at 28 weeks or later, with additional scans whenever clinically indicated; the highest grade recorded during the NICU stay was used. Because routine cranial ultrasonography was performed by the radiology department on weekdays during working hours, scans scheduled for a weekend or public holiday were performed on the next working day. Infants who did not undergo any cranial ultrasonography prior to death or discharge were classified as not evaluated for intraventricular hemorrhage (IVH). When severe IVH was analyzed as a separate outcome, infants who had not been evaluated were excluded from the denominator. In the composite outcome, infants who had not been evaluated and who died within 72 h were captured via the early-death component; no infants died after 72 h without having undergone cranial imaging. RDS was defined—independent of surfactant administration—by the coexistence of clinical signs of respiratory distress starting in the first hours of life (tachypnea, grunting, retractions, and increased oxygen requirement) and chest radiographic findings consistent with the condition (diffuse reticulogranular pattern with air bronchograms) [15]. BPD was assessed at 36 weeks PMA or at discharge home, whichever came first, in infants receiving supplemental oxygen for ≥28 days and was graded according to the National Institute of Child Health and Human Development (NICHD) criteria [16]: an oxygen requirement of <30% was considered moderate BPD, while a requirement for ≥30% oxygen and/or positive pressure support was considered severe BPD. hsPDA was defined, regardless of treatment, by the presence of hemodynamically significant clinical findings alongside echocardiographic criteria [ductal diameter ≥1.5 mm and/or left atrium/aortic root ratio ≥1.4 and the presence of a left-to-right shunt] [17]. NEC was defined as modified Bell stage ≥II [18]. PVL was defined as the presence of cystic periventricular leukomalacia on cranial ultrasonography. Air leak syndrome encompassed radiographically confirmed pneumothorax, pneumomediastinum, or pulmonary interstitial emphysema. Early- and late-onset sepsis were defined as sepsis commencing at ≤72 h and >72 h of life, respectively (definitions were based on positive blood culture or, in culture-negative cases, clinical signs accompanied by abnormal laboratory findings and ≥5 days of antibiotic therapy); fungal sepsis was defined as the growth of *Candida* species in blood culture [19]. Pulmonary hemorrhage was defined as the presence of bloody secretions from the endotracheal tube accompanied by clinical deterioration. ROP was classified according to the International Classification of Retinopathy of Prematurity. Inotrope use was defined as the administration of dopamine, dobutamine, or epinephrine/norepinephrine infusion within the first 7 days of life.

### 2.7. Statistical Analysis

Statistical analyses were performed using IBM SPSS Statistics version 22.0 (IBM Corp., Armonk, NY, USA) and Python 3 (statsmodels and SciPy libraries). Normality of continuous variables was assessed using the Shapiro–Wilk test. Variables with a normal distribution were presented as mean ± standard deviation (SD) and compared using Student’s *t*-test. Variables without a normal distribution were presented as median (interquartile range [IQR]) and compared using the Mann–Whitney U test. Categorical variables were expressed as *n* (%); comparisons utilized the Pearson chi-square test (with continuity correction) or Fisher’s exact test when the expected cell count was <5.

Multivariable logistic regression was used to estimate the association between delivery outside of working hours and the primary composite outcome, as well as the key secondary outcome of overall mortality. Overall mortality was modeled separately because it had the highest number of events (*n* = 100)—allowing for adequate adjustment—and was the most clinically significant outcome. Early mortality alone (21 events) had too low a frequency for full adjustment; therefore, it was analyzed adjusting only for gestational age. Model 1 included delivery outside of working hours as well as key confounding variables determined before or during birth—and thus unaffected by the exposure—namely gestational age, birth weight (per 100 g), cesarean section, antenatal steroids (complete course), and infant sex. Confounding variables were selected based on clinical validity and previous literature [3,9,20]; Cesarean section was included in the model due to baseline imbalances between the groups. Delivery room resuscitation was not included in the primary model because it occurred after the exposure was defined and could lie on the causal pathway between delivery timing, delivery room staffing/processes, and mortality (while also potentially serving as an indicator of underlying fetal distress); however, it was added to Model 2 to assess the extent to which the association was attenuated. Crude and adjusted odds ratios (OR and aOR) with 95% confidence intervals (CIs) are presented for all covariates. With 60 composite events and 100 deaths, there were at least 8.6 events per variable in each model. A two-sided *p*-value of <0.05 was considered statistically significant.

Several sensitivity analyses were performed. First, three infants born on public holidays during weekday working hours—initially grouped as “in-hours”—were reclassified as “out-of-hours.” Second, because certain perinatal variables were not recorded for 108 infants, the analysis was repeated using only those infants with complete perinatal data (*n* = 255). Third, to address incomplete IVH ascertainment, infants without any cranial ultrasound were classified as unassessed; the analysis of the composite outcome was repeated after excluding unassessed survivors and after classifying all unassessed infants as having the composite outcome (worst-case scenario), and severe IVH as a separate outcome was analyzed among assessed infants only.

Due to the retrospective design of the study, no a priori sample size calculation was performed; the precision of the estimates is indicated by 95% confidence intervals. To limit bias, predetermined inclusion and exclusion criteria were applied consistently. Furthermore, there were no significant changes in staffing arrangements or clinical protocols during the study period. Residual confounding arising from unmeasured variables (e.g., urgency and indication for delivery, disease severity scores, healthcare personnel experience, and concurrent workload) cannot be ruled out.

### 2.8. Declaration of Generative Artificial Intelligence Use

ChatGPT-6 (OpenAI, San Francisco, CA, USA) was used during the preparation of this manuscript to assist with English language editing, including correction of grammatical, spelling, and punctuation errors, formatting, and reference organization. ChatGPT was also used to assist in the preparation and refinement of the graphical abstract. All scientific content, data interpretation, conclusions, and final decisions regarding the manuscript were reviewed and verified by the authors, who take full responsibility for the content of the published work.

## 3. Results

During the study period (January 2021–May 2023), 512 preterm infants with a gestational age of <32 weeks were admitted to the neonatal intensive care unit (NICU) and evaluated for eligibility. Of these, 149 were excluded: 58 due to major congenital anomalies, 34 due to chromosomal anomalies, 27 due to perinatal asphyxia, 19 due to birth at another center, and 11 due to missing essential clinical data. The final cohort consisted of 363 infants; 160 (44.1%) were born during working hours, and 203 (55.9%) were born outside working hours (Figure 1). In addition, 17 infants born at <32 weeks of gestation died in the delivery room before NICU admission and were therefore not included in the study; 5 of these were born during working hours and 12 outside working hours (Figure 1).

Maternal and perinatal characteristics are presented in Table 1. No statistically significant differences were detected in maternal age or gravidity (*p* = 0.697 and *p* = 0.384, respectively). The rate of cesarean section was significantly higher in the in-hours group (88.1% vs. 78.8%, *p* = 0.028), consistent with routine scheduling of elective procedures during daytime hours. No statistically significant differences were detected in gestational diabetes, chorioamnionitis, preeclampsia, early membrane rupture, PPROM, multiple gestation, placental abruption, placenta previa, or antenatal steroid use.

Demographic and birth characteristics are summarized in Table 2. No statistically significant differences were detected in birth weight, gestational age, 5 min Apgar score, or sex distribution (all *p* > 0.05). The requirement for delivery-room resuscitation was significantly higher in the out-of-hours group (56.7% vs. 36.9%, *p* < 0.001).

Primary, secondary, and exploratory outcomes are presented in Table 3. The composite primary outcome (early death or severe IVH) occurred in 40 infants (19.7%) in the off-hours group and 20 infants (12.5%) in the on-hours group (*p* = 0.091). In the off-hours group, this outcome comprised 17 cases of early death and 25 cases of severe IVH (with 2 infants presenting both conditions), whereas in the on-hours group, it comprised 4 cases of early death and 16 cases of severe IVH (excluding overlaps). The overall mortality rate was higher in the off-hours group (32.5% vs. 21.3%; *p* = 0.023); the same applied to early deaths within the first 72 h (8.4% vs. 2.5%; *p* = 0.022, Fisher’s exact test). RDS was more frequent in the off-hours group (69.0% vs. 55.6%; *p* = 0.012). No statistically significant differences were found regarding severe IVH, air leak syndrome, hsPDA, NEC, or PVL. Among infants surviving to 36 weeks’ postmenstrual age (PMA), the incidence of moderate-to-severe BPD was 37.1% vs. 29.5% (*p* = 0.233), whereas the composite outcome of death or moderate-to-severe BPD was more frequent in the off-hours group (57.1% vs. 43.1%; *p* = 0.011). BPD assessment was conducted at 36 weeks of postmenstrual age (PMA) or at the time of discharge, whichever occurred first; no infants admitted to the neonatal intensive care unit (NICU) were transferred to another hospital. Accordingly, the group constituting the denominator for BPD calculations (132 infants during working hours, 143 infants outside working hours) includes all infants who were alive at 36 weeks PMA or who were discharged prior to this time point. In both groups, six infants passed away after 36 weeks PMA but before discharge; this accounts for the difference between the number of infants assessed for BPD and the number of infants discharged alive (126 and 137, respectively, as shown in Figure 1). In the composite endpoint of death or moderate-to-severe BPD, “death” refers to all deaths occurring prior to discharge; infants who had moderate-to-severe BPD and subsequently passed away (four infants during working hours, three infants outside working hours) were counted only once. The results remained consistent when the composite endpoint was restricted to death or moderate-to-severe BPD occurring before 36 weeks PMA (67/160 [41.9%] vs. 113/203 [55.7%]; *p* = 0.012). Exploratory outcomes included: No significant differences were found in the incidence of IVH (at any grade), pulmonary hemorrhage, surfactant administration, inotrope use during the first week, early- or late-onset sepsis, fungal sepsis, or ROP among infants who survived beyond 28 days. Surfactant was administered to 161 of 203 infants (79.3%) born outside of working hours and 118 of 160 infants (73.8%) born during working hours (*p* = 0.262); among infants with available data, initial respiratory support was invasive in 120 of 148 cases (81.1%) and 71 of 95 cases (74.7%), respectively (*p* = 0.309). While the majority of early-onset sepsis episodes were culture-negative clinical sepsis (culture-confirmed: 10 [4.9%] and 8 [5.0%]), culture-confirmed late-onset sepsis occurred in 85 (41.9%) and 70 (43.8%) infants, respectively, with staphylococci identified in 83% of culture-positive episodes.

Nutritional outcomes, duration of IMV, and length of hospital stay are presented in Table 4. The time to achieve full enteral and full oral feeding, as well as the length of hospital stay, were analyzed for infants discharged alive; the duration of IMV was analyzed for infants who received IMV (in both the overall group and among survivors), and no statistically significant difference was found. Given the higher mortality rate during off-hours, these comparisons among survivors should not be interpreted as evidence of a similar or better clinical course in that group.

Umbilical cord blood gas parameters are shown in Table 5; no statistically significant differences were detected.

Crude and adjusted associations are presented in Table 6. Regarding the primary composite outcome, birth outside of working hours did not show a significant association after adjustment for initial confounders (Model 1 aOR 1.46; 95% CI 0.77–2.77; *p* = 0.242) or after additional adjustment for delivery room resuscitation (Model 2 aOR 1.24; 95% CI 0.64–2.39; *p* = 0.526); the primary determinant was lower gestational age (Model 1 aOR 0.53 [per week]; 95% CI 0.40–0.70; *p* < 0.001). Regarding overall mortality, birth outside of working hours was associated with higher odds in Model 1 (aOR 1.88; 95% CI 1.11–3.20; *p* = 0.020); however, this estimate attenuated and lost statistical significance upon additional adjustment for delivery room resuscitation (Model 2 aOR 1.58; 95% CI 0.91–2.75; *p* = 0.101). In Model 1, a complete course of antenatal steroids (aOR 0.50; 95% CI 0.29–0.87; *p* = 0.015) and higher birth weight (aOR 0.86 [per 100 g]; 95% CI 0.75–0.97; *p* = 0.019) were associated with lower mortality; In Model 2, resuscitation in the delivery room was associated with higher mortality (aOR 3.40; 95% CI 1.84–6.27; *p* < 0.001). No association was found between cesarean delivery and either outcome. The likelihood of early death was higher outside of standard working hours (crude OR 3.56; 95% CI 1.17–10.81); a similar, albeit less precise, estimate was obtained after adjusting for gestational age (adjusted OR 3.07; 95% CI 0.98–9.61; *p* = 0.053). The initial cranial ultrasound was performed at a median of 3 days (interquartile range [IQR] 2–4) in both groups. Among infants born during working hours, 85 (53.1%) underwent imaging within the first 72 h, 70 between days 4 and 7, and 1 after day 7; among those born outside working hours, 129 (63.5%) underwent imaging within the first 72 h, 63 between days 4 and 7, and 2 after day 7. Delays in the first scan were mainly related to the scheduled day falling on a weekend or public holiday; when day 3 fell on a weekend, the first scan was performed after 72 h in 66 of 104 infants (63.5%), compared with 70 of 246 (28.5%) otherwise. Twelve infants could not be evaluated for intraventricular hemorrhage (IVH) (4 in-hours and 8 out-of-hours); 10 of these infants died within the first 72 h before any imaging could be performed (3 and 7 infants, respectively), while 2 infants survived, but no imaging records could be located for them (1 infant from each group). There were no deaths occurring after the first 72 h in which no cranial imaging had been performed. Among the infants who could be evaluated, severe IVH was detected in 16/156 (10.3%) of in-hours and 25/195 (12.8%) of out-of-hours infants (*p* = 0.565). Sensitivity analyses—conducted either by excluding unassessed survivors or by assuming that all such infants reached the composite endpoint—yielded results consistent with the primary analysis (Table 7).

In sensitivity analyses (Table 7), reclassifying three births that occurred on holidays yielded a similar mortality estimate (adjusted OR 1.73; 95% CI 1.02–2.94); when the analysis was restricted to infants with complete perinatal data, a similar point estimate with a wider confidence interval (CI) was obtained (adjusted OR 1.70; 95% CI 0.90–3.20). Excluding unassessed survivors (aOR 1.46; 95% CI 0.77–2.77) or classifying all unassessed infants as having the composite outcome (aOR 1.41; 95% CI 0.76–2.62) did not materially change the composite estimate, and severe IVH among assessed infants was not associated with out-of-hours birth (aOR 1.14; 95% CI 0.56–2.34). Estimates regarding the composite outcome were not statistically significant in any of the sensitivity analyses.

## 4. Discussion

This study examined the relationship between the timing of birth (during vs. outside of working hours) and early and intermediate neonatal outcomes in infants born before 32 weeks of gestation. The key findings are as follows: (1) birth outside of working hours was not significantly associated with the primary composite outcome of early mortality or severe intraventricular hemorrhage (IVH); (2) overall and early mortality rates were higher among infants born outside of working hours; (3) the association with overall mortality persisted after adjustment for baseline confounders but weakened and lost statistical significance after additional adjustment for delivery room resuscitation, a potential mediating variable; and (4) delivery room resuscitation and RDS occurred more frequently in births outside of working hours. No statistically significant differences were found regarding most major neonatal morbidities.

Preterm birth, particularly before 32 weeks, remains one of the most important determinants of neonatal mortality and morbidity. Owing to pulmonary immaturity, cerebral vascular fragility, and an underdeveloped immune system, these infants are at increased risk for complications such as RDS, IVH, and BPD. Birth timing has therefore been considered a potential factor influencing outcomes, particularly because of possible differences in healthcare organization, staffing, and resource availability [13].

Previous studies examining birth timing and neonatal outcomes have reported conflicting results. Large cohort analyses have demonstrated an increased risk of death or severe IVH among very low birth weight infants delivered during nighttime hours, particularly in settings with reduced overnight staffing [9]. However, more recent studies indicate that this association may largely be explained by differences in case-mix, illness severity, or healthcare-system characteristics, and that birth timing itself may not represent an independent risk factor once appropriate adjustments are performed [10]. In our cohort, the association between delivery outside of standard working hours and mortality persisted after adjustment for baseline characteristics but attenuated after adjustment for delivery room resuscitation; thus, our data are consistent with both a modest effect of delivery timing and residual confounding due to case mix [10,13].

Gestational age was the strongest predictor of the composite outcome (aOR 0.53 per week), and birth weight was independently associated with mortality (aOR 0.86 per 100 g). The weaker independent association of gestational age with mortality in the adjusted model likely reflects its strong correlation with birth weight. These findings are consistent with large epidemiological studies showing that maturity is the strongest predictor of prognosis in preterm infants, as increasing gestational age is associated with maturation of pulmonary, neurological, and metabolic systems [1,2,20].

Antenatal steroid administration was also identified as an independent protective factor (aOR 0.50, 95% CI 0.29–0.87). Antenatal corticosteroids accelerate fetal lung maturation by enhancing surfactant production and reducing the incidence of RDS and its complications [3,4]. Multiple large randomized trials and systematic reviews have consistently demonstrated that antenatal corticosteroids significantly reduce neonatal mortality, severe respiratory complications, and IVH [4,21].

Resuscitation performed in the delivery room showed a strong association with mortality (Model 2 aOR 3.40). The need for intubation or CPR at birth may reflect more severe perinatal distress, extreme prematurity, or acute intrapartum events [9,11]; however, since resuscitation occurs after birth and its administration may depend on the personnel present, it could also play a role in the causal pathway between birth timing and mortality. The attenuation of the estimate for births outside standard working hours (from aOR 1.88 to 1.58) upon adjustment for the resuscitation variable is consistent with the hypothesis that the association is partly explained by events in the delivery room (partial mediation), but also with the possibility that resuscitation serves as an indicator of underlying fetal distress; our data do not allow us to distinguish between these explanations.

The absence of statistically significant differences regarding major morbidities (IVH, BPD in survivors, NEC, hsPDA) should be interpreted with caution: the study lacked sufficient statistical power for these outcomes, and the morbidities assessed in survivors are influenced by the higher mortality rate in the group born outside standard working hours; indeed, the higher rate of death or BPD among births outside standard working hours (57.1% vs. 43.1%) illustrates this.

Increasing attention has been directed toward the role of healthcare-organizational factors in neonatal outcomes. Salazar et al. demonstrated that NICU capacity strain and high patient-to-staff ratios are associated with increased risks of mortality and morbidity in preterm infants [11], highlighting that temporal effects of birth timing must be interpreted within the broader context of healthcare-system organization.

Regarding external validity, these findings should be interpreted with caution in settings where staffing models, specialist availability, or care protocols differ substantially from ours. Our NICU operated with attending-level neonatology support during daytime hours and resident/specialist on-call coverage with phone consultation at night—a model common in middle-income-country tertiary centers. Institutions with 24 h in-house neonatologist coverage, or conversely those with more limited overnight resources, may observe different magnitudes of the off-hours effect. The relatively high overall mortality in our cohort (27.5%) compared with high-income-country benchmarks may reflect population-level differences in gestational-age distribution, antenatal-care access, and case severity, further limiting direct comparability.

A notable finding was that the early mortality rate within the first 72 h was higher for births occurring outside of regular working hours (8.4% vs. 2.5%; *p* = 0.022), accompanied by an increased rate of delivery room resuscitation (56.7% vs. 36.9%; *p* < 0.001). This situation can be interpreted in two ways: births occurring outside regular hours may involve infants experiencing more severe perinatal compromise, or initial stabilization processes outside standard working hours may differ. Our data do not allow for a definitive distinction between these two possibilities. Key baseline indicators—such as gestational age, birth weight, 5 min Apgar score, and umbilical cord pH and base excess—did not show significant differences between the groups. This weakens the hypothesis that a major difference in baseline status or the level of intrapartum distress is the sole explanation. However, data on the urgency and indication for delivery—factors potentially associated with both out-of-hours delivery and the need for resuscitation—were not recorded. Factors such as the duration of stabilization, the composition and experience of the delivery room team, and workload were also not measured. Consequently, the higher resuscitation rate may reflect differences in delivery room practices, such as less experienced teams employing lower thresholds for intubation, unmeasured variations in case distribution, or a combination of both. Studies on the “weekend effect” have demonstrated that the immediate postpartum period is the timeframe most affected by variations in staffing [7,9]. Given the low number of early deaths (*n* = 21), this finding should be considered hypothesis-generating and warrants prospective studies that document delivery room processes.

A higher rate of RDS was observed in births occurring outside of regular working hours (69.0% vs. 55.6%; *p* = 0.012), despite similar rates of antenatal steroid exposure (35.0% vs. 31.9%; *p* = 0.611) and comparable gestational ages. The low overall rate of documented complete antenatal steroid courses may reflect a high proportion of unplanned deliveries; however, as the urgency and indication for delivery were not recorded, this interpretation remains speculative. No significant differences were found regarding surfactant administration (161/203 [79.3%] vs. 118/160 [73.8%], *p* = 0.262) or the initiation of invasive respiratory support (120/148 [81.1%] vs. 71/95 [74.7%], *p* = 0.309). Since the timing of surfactant administration and the quality of respiratory management were not measured, we cannot determine whether the difference in RDS rates stemmed from case distribution, diagnostic practices, or care processes. In a large Chinese cohort study, Chen et al. reported that births occurring outside of regular working hours were associated with a higher incidence of RDS and a more frequent need for surfactant [8].

Infectious complications were analyzed as exploratory outcomes, and no significant difference was observed between the groups. The high rates of early-onset (28.9%) and late-onset (65.3%) sepsis largely reflect our definitions: while the majority of early-onset cases were culture-negative clinical sepsis (with a culture-proven early-onset sepsis rate of 5.0%), culture-proven late-onset sepsis was identified in 42.7% of the overall study population (*n* = 363), predominantly involving staphylococcal species.

The frequent diagnosis of clinical sepsis in extremely preterm infants presenting with non-specific symptoms, combined with the burden of healthcare-associated infections in our unit, indicates a need to strengthen infection prevention measures and antibiotic stewardship, regardless of the time of birth. Adherence to central catheter use, antifungal prophylaxis, and infection prevention protocols was not assessed; therefore, the rate of fungal sepsis—which was based on small absolute numbers and appeared numerically higher outside of working hours (4.4% vs. 1.3%, *p* = 0.121)—cannot be attributed to differences in care practices.

Overall, while our findings do not support a strong impact of birth timing on the composite outcome of early death or severe intraventricular hemorrhage (IVH), they do point to a higher mortality rate among infants born outside of working hours that cannot be explained by baseline characteristics. Births occurring outside of working hours may disproportionately include emergency and unplanned deliveries associated with higher perinatal risk; however, as our data lack information regarding the urgency of delivery, indication for birth, and acute fetal distress, this represents a potential source of residual confounding rather than a definitive explanation. The higher early mortality and resuscitation burden observed outside of standard working hours indicate that delivery room preparedness and care during the first hours of life warrant further prospective evaluation.

### Limitations

This study has several limitations. First, the retrospective and single-center design precludes causal inference and may limit generalizability. Second, data regarding planned versus emergency delivery, spontaneous versus indicated preterm birth, indications for delivery, and acute fetal distress were unavailable; mode of delivery is an inadequate proxy for these factors, making residual confounding due to the urgency of delivery a primary concern. Third, the mechanisms underlying the observed differences could not be investigated because factors such as staffing levels during delivery, healthcare provider experience, workload, stabilization time, and the timing of interventions like surfactant administration were not measured. Fourth, illness severity scores (e.g., CRIB-II or SNAP-II) were not consistently available, limiting risk stratification beyond gestational age and birth weight. Fifth, the exclusion of infants with perinatal asphyxia (*n* = 27) may have introduced selection bias, as intrapartum distress can be associated with both the timing of delivery and mortality; this may have biased our estimates, most likely toward the null, and a sensitivity analysis including these infants was not feasible. Sixth, the study lacked the statistical power to detect moderate differences in the composite or secondary outcomes, and the number of early deaths was low.

## 5. Conclusions

In this single-center retrospective cohort study of infants born before 32 weeks of gestation, delivery outside of standard working hours was not found to be significantly associated with the composite primary outcome of early death or severe IVH; however, it was associated with overall mortality, early death, delivery room resuscitation, and RDS. The association with mortality persisted after adjustment for baseline characteristics but weakened and became inconclusive following additional adjustment for delivery room resuscitation, while remaining consistent with a clinically significant increase in risk. Prospective multicenter studies should evaluate the timing of delivery in conjunction with indications for delivery, staffing levels, workload, and metrics of delivery room processes.

## Figures and Tables

**Table 1:** Comparison of perinatal characteristics of the study population.

Variable	In-Hours (*n* = 160)	Out-of-Hours (*n* = 203)	*p*-Value
Maternal age (years), median (IQR)	28.0 (24.8–34.0)	29.0 (24.0–33.0)	0.697
Gravidity, median (IQR)	3.0 (1.0–4.0)	3.0 (1.0–5.0)	0.384
Cesarean section, *n* (%)	141 (88.1)	160 (78.8)	0.028
Gestational diabetes, *n* (%)	3 (1.9)	5 (2.5)	1.000
Chorioamnionitis, *n* (%)	3 (1.9)	9 (4.4)	0.241
Preeclampsia, *n* (%)	13 (8.1)	16 (7.9)	1.000
Early membrane rupture, *n* (%)	27 (16.9)	33 (16.3)	0.988
PPROM, *n* (%)	13 (8.1)	13 (6.4)	0.670
Antenatal steroid use (complete course), *n* (%)	51 (31.9)	71 (35.0)	0.611
Multiple gestation, *n* (%)	46 (28.8)	42 (20.7)	0.098
Placental abruption, *n* (%)	6 (3.8)	11 (5.4)	0.619
Placenta previa, *n* (%)	0 (0.0)	3 (1.5)	0.258 †

PPROM, preterm premature rupture of membranes; IQR, interquartile range. Values are median (IQR) for continuous variables and *n* (%) for categorical variables. *p*-values were calculated using the Mann–Whitney U test, the chi-square test with continuity correction, or the Fisher exact test (†), as appropriate. Planned versus emergency delivery, delivery indication, spontaneous versus indicated preterm birth, and acute fetal compromise were not consistently recorded and could not be analyzed.

**Table 2:** Demographic and birth characteristics of the study population.

Variable	In-Hours (*n* = 160)	Out-of-Hours (*n* = 203)	*p*-Value
Birth weight (g), mean ± SD	1138.3 ± 393.2	1155.9 ± 405.0	0.675
Gestational age (weeks), mean ± SD	28.24 ± 2.39	27.84 ± 2.50	0.127
5 min Apgar score, median (IQR)	7.0 (6.0–8.0)	7.0 (6.0–7.8)	0.522
Male sex, *n* (%)	83 (51.9)	111 (54.7)	0.670
Delivery-room resuscitation, *n* (%)	59 (36.9)	115 (56.7)	<0.001

SD, standard deviation; IQR, interquartile range.

**Table 3:** Primary and secondary clinical outcomes.

Variable	In-Hours (*n* = 160)	Out-of-Hours (*n* = 203)	*p*-Value
***Primary outcome***			
Early mortality (≤72 h) or severe IVH (grades III–IV), *n* (%)	20 (12.5)	40 (19.7)	0.091
***Prespecified secondary outcomes***			
Overall in-hospital mortality, *n* (%)	34 (21.3)	66 (32.5)	0.023
Early mortality (≤72 h), *n* (%)	4 (2.5)	17 (8.4)	0.022 †
Severe IVH (grades III–IV), *n*/N (%) *	16/156 (10.3)	25/195 (12.8)	0.565
RDS, *n* (%)	89 (55.6)	140 (69.0)	0.012
Air-leak syndrome, *n* (%)	5 (3.1)	8 (3.9)	0.896
hsPDA, *n* (%)	67 (41.9)	87 (42.9)	0.935
NEC (modified Bell stage ≥II), *n* (%)	22 (13.8)	25 (12.3)	0.805
Moderate-to-severe BPD, *n*/N alive at 36 weeks PMA or discharged home earlier (%)	39/132 (29.5)	53/143 (37.1)	0.233
Death or moderate-to-severe BPD, *n* (%)	69 (43.1)	116 (57.1)	0.011
PVL, *n*/N surviving >72 h (%)	15/156 (9.6)	23/186 (12.4)	0.527
***Exploratory outcomes***			
Any-grade IVH, *n* (%)	38 (23.8)	58 (28.6)	0.361
Pulmonary hemorrhage, *n* (%)	11 (6.9)	19 (9.4)	0.508
Surfactant administration, *n* (%)	118 (73.8)	161 (79.3)	0.262
Initial invasive ventilation, *n*/N with available data (%)	71/95 (74.7)	120/148 (81.1)	0.309
Inotrope use in the first week, *n* (%)	27 (16.9)	39 (19.2)	0.663
Early-onset sepsis, *n* (%)	47 (29.4)	58 (28.6)	0.959
Culture-proven, *n* (%)	8 (5.0)	10 (4.9)	1.000
Clinical (culture-negative), *n* (%)	39 (24.4)	48 (23.6)	0.970
Late-onset sepsis, *n* (%)	112 (70.0)	125 (61.6)	0.118
Culture-proven, *n* (%)	70 (43.8)	85 (41.9)	0.801
Clinical (culture-negative), *n* (%)	42 (26.3)	40 (19.7)	0.176
Fungal sepsis, *n* (%)	2 (1.3)	9 (4.4)	0.121 †
ROP (any stage), *n*/N surviving >28 days (%)	8/139 (5.8)	9/155 (5.8)	1.000

IVH, intraventricular hemorrhage; RDS, respiratory distress syndrome; hsPDA, hemodynamically significant patent ductus arteriosus; NEC, necrotizing enterocolitis; BPD, bronchopulmonary dysplasia; PMA, postmenstrual age; PVL, periventricular leukomalacia; ROP, retinopathy of prematurity. Values are *n* (%) of the whole group unless a denominator (*n*/N) of eligible survivors is shown. *p*-values were calculated using the chi-square test with continuity correction or the Fisher exact test (†). In the out-of-hours group, two infants had both early death and severe IVH; there was no overlap in the in-hours group. Initial respiratory support was analyzed in infants with available data. Culture-proven sepsis was defined by growth of a pathogen in blood culture, and clinical sepsis by clinical and laboratory findings with a negative blood culture. * Unassessed infants (*n* = 4 and *n* = 8) were excluded from the denominator.

**Table 4:** Nutritional outcomes, invasive mechanical ventilation, and length of stay.

Variable	In-Hours (*n* = 160)	Out-of-Hours (*n* = 203)	*p*-Value
Age at start of enteral feeding (days) ^1^	2.0 (1.0–3.0) [*n* = 94]	2.0 (1.0–3.0) [*n* = 145]	0.336
Feeding intolerance, n/N (%) ^1^	66/95 (69.5)	98/148 (66.2)	0.597
Time to full enteral feeding (days) ^2^	20.0 (10.0–33.0) [*n* = 65]	16.5 (10.0–30.0) [*n* = 84]	0.461
Time to full oral feeding (days) ^2^	48.0 (29.3–71.8) [*n* = 66]	43.0 (30.0–66.0) [*n* = 89]	0.676
Length of hospital stay (days) ^2^	45.0 (29.0–69.0) [*n* = 126]	45.0 (31.0–69.0) [*n* = 137]	0.698
IMV duration (days); infants receiving IMV ^3^	6.0 (2.0–17.5) [*n* = 79]	5.0 (2.0–18.8) [*n* = 130]	0.293
IMV duration (days); survivors receiving IMV ^3^	6.0 (2.0–16.5) [*n* = 58]	4.0 (1.5–17.5) [*n* = 83]	0.611

IMV, invasive mechanical ventilation; IQR, interquartile range. Values are median (IQR) with the number of infants analyzed [*n*] unless otherwise stated; *p*-values were calculated using the Mann–Whitney U test. ^1^ Infants with available data. ^2^ Infants discharged alive (126 in-hours, 137 out-of-hours) with available data; deaths are excluded from these analyses. ^3^ Infants who received IMV, overall and among survivors. Because mortality was higher out-of-hours, survivor-based comparisons should be interpreted with caution.

**Table 5:** Umbilical cord blood gas parameters.

Variable	In-Hours (*n* = 160)	Out-of-Hours (*n* = 203)	*p*-Value
Umbilical cord pH	7.31 (7.23–7.36)	7.29 (7.22–7.35)	0.127
Umbilical cord base excess (mmol/L)	−5.25 (−7.70 to −2.80)	−5.30 (−8.55 to −2.90)	0.655

Values are median (IQR). Umbilical cord samples were venous. *p*-values were calculated using the Mann–Whitney U test.

**Table 6:** Logistic regression analysis of factors associated with the primary composite outcome and overall mortality.

Variable	Crude OR (95% CI)	*p*-Value	Model 1 aOR (95% CI)	*p*-Value	Model 2 aOR (95% CI)	*p*-Value
***A. Primary composite outcome: early mortality or severe IVH (60 events)***						
Out-of-hours birth (vs. in-hours)	1.72 (0.96–3.08)	0.069	1.46 (0.77–2.77)	0.242	1.24 (0.64–2.39)	0.526
Gestational age (per week)	0.62 (0.53–0.71)	<0.001	0.53 (0.40–0.70)	<0.001	0.57 (0.44–0.76)	<0.001
Birth weight (per 100 g)	0.79 (0.72–0.87)	<0.001	1.12 (0.96–1.31)	0.161	1.13 (0.96–1.32)	0.136
Cesarean section	0.62 (0.32–1.21)	0.162	1.15 (0.50–2.61)	0.741	1.03 (0.45–2.35)	0.942
Antenatal steroids (complete course)	0.90 (0.50–1.63)	0.727	0.78 (0.41–1.48)	0.441	0.59 (0.30–1.16)	0.128
Male sex (vs. female)	0.85 (0.49–1.48)	0.559	0.88 (0.48–1.63)	0.689	0.97 (0.52–1.81)	0.918
Delivery-room resuscitation	6.34 (3.17–12.68)	<0.001	—		3.43 (1.56–7.56)	0.002
***B. Overall in-hospital mortality (100 events)***						
Out-of-hours birth (vs. in-hours)	1.79 (1.11–2.88)	0.018	1.88 (1.11–3.20)	0.020	1.58 (0.91–2.75)	0.101
Gestational age (per week)	0.71 (0.64–0.79)	<0.001	0.85 (0.70–1.02)	0.081	0.92 (0.76–1.12)	0.429
Birth weight (per 100 g)	0.80 (0.74–0.86)	<0.001	0.86 (0.75–0.97)	0.019	0.86 (0.76–0.98)	0.028
Cesarean section	0.76 (0.42–1.37)	0.363	0.91 (0.45–1.83)	0.789	0.86 (0.43–1.74)	0.681
Antenatal steroids (complete course)	0.61 (0.37–1.02)	0.060	0.50 (0.29–0.87)	0.015	0.36 (0.20–0.65)	<0.001
Male sex (vs. female)	1.36 (0.86–2.17)	0.191	1.59 (0.95–2.67)	0.078	1.73 (1.02–2.95)	0.043
Delivery-room resuscitation	4.34 (2.61–7.20)	<0.001	—		3.40 (1.84–6.27)	<0.001

OR, odds ratio; aOR, adjusted odds ratio; CI, confidence interval. Crude ORs were estimated by univariable logistic regression. Model 1 included out-of-hours birth, gestational age, birth weight, cesarean section, antenatal steroids, and sex (baseline confounders). Model 2 additionally included delivery-room resuscitation (intubation, cardiopulmonary resuscitation, or epinephrine), a potential mediator. All 363 infants were included in each model.

**Table 7:** Sensitivity analyses for the association between out-of-hours birth and outcomes.

Analysis	*n* (In-Hours/Out-of-Hours)	Composite Outcome aOR (95% CI)	*p*-Value	Overall Mortality aOR (95% CI)	*p*-Value
Primary analysis (Model 1)	363 (160/203)	1.46 (0.77–2.77)	0.242	1.88 (1.11–3.20)	0.020
Primary analysis + delivery-room resuscitation (Model 2)	363 (160/203)	1.24 (0.64–2.39)	0.526	1.58 (0.91–2.75)	0.101
Holiday births reclassified as out-of-hours	363 (157/206)	1.32 (0.70–2.50)	0.392	1.73 (1.02–2.94)	0.042
Infants with fully documented perinatal data	255 (101/154)	1.33 (0.67–2.66)	0.419	1.70 (0.90–3.20)	0.102
Unassessed survivors excluded ^a^	361 (159/202)	1.46 (0.77–2.77)	0.243	1.88 (1.10–3.19)	0.020
All unassessed infants classified as having the composite outcome ^b^	363 (160/203)	1.41 (0.76–2.62)	0.281	—	—
Severe IVH as a separate outcome, assessed infants only ^c^	351 (156/195)	1.14 (0.56–2.34) ^c^	0.719	—	—

aOR, adjusted odds ratio for out-of-hours versus in-hours birth; CI, confidence interval. Unless stated otherwise, estimates are adjusted for the Model 1 covariates (gestational age, birth weight, cesarean section, antenatal steroids, and sex). IVH, intraventricular hemorrhage. Infants who did not undergo cranial ultrasonography prior to death or discharge were classified as not assessed for IVH (*n* = 4 for births during working hours, *n* = 8 for births outside working hours); of these, ten died within the first 72 h and were thus accounted for within the early mortality component, while two survived. ^a^ The two infants who were not assessed and survived (one from each group) were excluded from the analysis. ^b^ All 12 infants who were not assessed were assumed to have experienced the composite outcome (worst-case scenario); the mortality estimate remains the same as in the primary analysis. ^c^ This estimate pertains to severe IVH (grades III–IV) rather than the composite outcome; infants who were not assessed have been excluded from the denominator.

## Data Availability

The datasets analyzed during the current study are available from the corresponding author upon reasonable request. The data generated and/or analyzed in this study are presented within the article.

## Abbreviations

The following abbreviations are used in this manuscript:

aOR	Adjusted odds ratio
BPD	Bronchopulmonary dysplasia
C/S	Cesarean section
CI	Confidence interval
CPR	Cardiopulmonary resuscitation
hsPDA	Hemodynamically significant patent ductus arteriosus
IMV	Invasive mechanical ventilation
IQR	Interquartile range
IVH	Intraventricular hemorrhage
NEC	Necrotizing enterocolitis
NICHD	National Institute of Child Health and Human Development
NICU	Neonatal intensive care unit
NSD	Normal spontaneous vaginal delivery
OR	Odds ratio
PMA	Postmenstrual age
PPROM	Preterm premature rupture of membranes
PVL	Periventricular leukomalacia
RDS	Respiratory distress syndrome
ROP	Retinopathy of prematurity
SD	Standard deviation
